# *Basilaphelenchus hyrcanus* n. sp. (Rhabditida: Tylaphelenchinae) associated with bark of a beech tree (*Fagus orientalis* Lipsky) from northern Iran

**DOI:** 10.21307/jofnem-2021-028

**Published:** 2021-03-17

**Authors:** Behrouz Golhasan, Esmaeil Miraeiz, Zahra Tanha maafi, Ramin Heydari

**Affiliations:** 1 Department of Plant Protection, College of Agriculture and Natural resources, University of Tehran, Karaj, Iran; 2 Iranian Research Institute of Plant Protection, Agricultural Research, Education and Extension Organization (AREEO), Tehran, Iran

**Keywords:** LSU, Golestan province, Molecular, Morphology, New species, Phylogeny, SSU, Taxonomy

## Abstract

*Basilaphelenchus hyrcanus* n. sp., the sixth species of the newly established genus was isolated during a nematode survey associated with bark samples of beech tree in northern Iran, which differs from the other species by body size, stylet length, metacorpus structure, and tail characters of both genders. The new species is also characterized by elevated cephalic region with sclerotised vestibule, posteriorly directed stylet knobs, well-developed metacorpus filling corresponding body region, position of excretory pore at the level of base of metacarpus, length of post uterine sac by 38–60 μm long, conoid elongate tail by sharp to finely rounded tip in female. Based upon the morphological characteristics and phylogenetic analyses of partial 18 S and D2-D3 28 S rDNA segments, the new species mostly resembles *B. magnabulbus*. However, *B. hyrcanus* n. sp. is clearly different from this species by having a longer stylet, different position of the excretory pore, a different male tail tip characters and 4.4 and 13.2% sequence divergences in 18 S and D2-D3 28 S, respectively.

Aphelenchoididae Skarbilovich, 1947 is a diverse family that includes fungal feeders, plant parasites, insect parasites and predators (Hunt, 1993, 2008). The family is separated into six subfamilies, namely Acugutturinae Hunt, 1980; Aphelenchoidinae Skarbilovich, 1947; Ektaphelenchinae Paramonov, 1964; Entaphelenchinae Nickle, 1970; Parasitaphelenchinae Rühm, 1956, and Seinurinae Husain and Khan, 1967. The molecular phylogenetic relationships of Aphelenchoididae inferred from 18 S and 28 S D2-D3 of rDNA by Kanzaki and Giblin-Davis (2012) showed four major clades. Based on molecular data, Kanzaki et al. (2014) erected a new subfamily Tylaphelenchinae including *Pseudaphelenchus* Kanzaki et al., 2009 and *Tylaphelenchus* Rühm, 1956 assigning them to clade one. Recently, Pedram et al. (2018) added a new genus, *Basilaphelnchus* Pedram et al., 2018, to this subfamily. They also considered *Albiziaphelenchus* Bajaj, 2012 as the fourth genus for Tylaphelenchinae. However, molecular data are available only for *Pseudaphelenchus* and *Basilaphelenchus.*

*Basilaphelenchus* currently consists of five nominal species of which four species have been proposed from Iran, namely *B. persicus* Pedram et al., 2018, *B. brevicaudatus* Mirzaie Fouladvand et al., 2019a, *B. gorganensis* Mirzaie Fouladvand et al., 2019b, and *B. magnabulbus* Aliramaji et al., 2019a. In a nematode survey in the north of Iran, an aphelenchid nematode resembling the genus *Basilaphelenchus* was recovered from bark samples collected from a beech tree (*Fagus orientalis* Lipsky) in Aliabad-e-Katul, Golestan province. The isolated population did not match with any of the described species of *Basilaphelenchus* and was revealed to be a new species that is illustrated and described herein as *B. hyrcanus* n. sp. Based on partial 18 S and 28 S D2-D3 rDNA sequences, the phylogenetic relationships with other genera of Aphelenchoididae are discussed.

The objectives of the present study were to (a) characterize the new species by the morphological features (b) determine the molecular phylogenetic affinities of *B. hyrcanus* n. sp. with closely related species using both partial 18 S and 28 S D2-D3 rDNA sequences.

## Materials and methods

### Sampling, extraction, mounting, and drawing

Several wood and bark samples were collected from Aliabad-e-Katul, Golestan province, Iran, during September to December 2019. The samples were placed in plastic bags, transferred to the Nematology Laboratory of University of Tehran and maintained at 4°C. Nematodes were extracted from bark samples by the tray method (Whitehead and Hemming, 1965). For the morphological study some nematodes were mounted in tap water and examined immediately. Others were killed and fixed using hot 4% formaldehyde solution and processed to pure glycerin according to De Grisse (1969). Permanent slides were prepared and nematodes observed using a light microscope (Nikon E200). Drawings were made using a drawing tube attached to the same microscope. Photographs of live nematodes were taken with a digital camera attached to the microscope.

### Nematode culture

In order to confirm the mycophagy behavior of the populations, as well as for purification of the samples, the populations were cultured according to Adeldoost et al. (2016). In all, 20 ml of potato dextrose agar was added to 9 cm diam. plastic petri dishes. Then fresh subcultures of *Botrytis cinerea* were prepared and kept in an incubator at 25°C in the dark. About 10 days later, five female nematodes were added to Petri dishes with fungus. The plates were examined after 20 days.

### DNA extraction, PCR, and sequencing

Nematode DNA was extracted from a single individual and PCR performed as described by Golhasan et al. (2016). A single live nematode was selected, examined on a temporary slide and transferred to a small drop of TE buffer (10 mM Tris-Cl, 0.5 mM EDTA; pH 9.0, Qiagen) on a clean slide and cut into small pieces using a sterilised razor blade. The suspension was collected by adding 20 µl AE Buffer and 2 µl proteinase K (600 µg/ml) (Promega, Benelux, the Netherlands). The tubes were incubated at 65°C for 1 h, 95°C for 15 min, and 80°C for 15 min. In all, 1 µl of extracted DNA was transferred to an Eppendorf tube containing: 2.5 µl 10X NH4 reaction buffer, 0.75 µl MgCl2 (50 mM), 0.25 µl dNTPs mixture (10 mM each), 0.75 µl of each primer (10 mM), 0.2 µl BIOTAQ DNA Polymerase (Bioline, UK), and ddH2O to a final volume of 25 µl. Two sets of primers (synthesised by Invitrogen) were used to amplify the partial 18 S and 28 S D2-D3 region of rDNA. Primers for the D2-D3 domain of 28 S rDNA were D2A (forward: 5’–ACA AGT ACC GTG AGG GAA AGT TG–3’) and D3B (reverse: 5’–TCG GAA GGA ACC AGC TAC TA–3’) (De Ley et al., 1999). Primers for amplification of partial SSU rDNA were forward primer 1096 F (5’–GGT AAT TCT GGA GCT AAT AC–3’) and reverse primer 1912R (5’–TTT ACG GTC AGA ACT AGG G–3’) (Holterman et al., 2006). The thermal cycling program was as follows: denaturation at 95°C for 2 min, followed by 35 cycles of denaturation at 94°C for 30 s, annealing at 55°C for 40 s, extension at 72°C for 80 s. A final extension step was performed at 72°C for 10 min. This program was used for amplification of both genomic regions. PCR products were separated on 1% agarose gels and visualized by staining with ethidium bromide. The PCR products were sequenced in both directions using the same primers with an ABI 3730XL sequencer (Bioneer Corporation, South Korea). Sequences were deposited in the GenBank database under accession numbers MN888475 (D2-D3 region of the 28 S rDNA gene) and MN888474 (18 S rDNA gene).

### Phylogenetic analyses

The obtained partial 18 S and partial 28 S D2-D3 sequences of rDNA gene of *Basilaphelenchus hyrcanus* n. sp. were compared with those of other aphelenchid species available in GenBank using the BLAST homology search program. The alignment of selected sequences was conducted using the MAFFT version 7 (http://mafft.cbrc.jp/alignment/server/) (Katoh and Standley, 2013). After manually trimming the alignment, the Gblocks program (version 0.91b) using all three less stringent parameters, a server tool at the Castresana Lab (http://molevol.cmima.csic.es/castresana/Gblocks_server.html), was used to eliminate poorly aligned regions or divergent positions. The best fitted model of DNA evolution with the base frequency, the proportion of invariable sites, the gamma distribution shape parameters and substitution rates was selected using MrModeltest 2 (Nylander, 2004). Under the Akaike Information Criterion (AIC), GTR + I + G model was selected for both partial 18 S and partial 28 S D2-D3 regions. Bayesian inference (BI) analysis for each gene was obtained separately using MrBayes 3.2.3 (Ronquist and Huelsenbeck, 2003) with four chains (three heated and one cold). The number of generations for the total analysis was set to 2 × 10^6^, with the chain sampled every 1,000 generations. After discarding burnin samples and evaluating convergence, the remaining samples were retained for further analyses. The Markov chain Monte Carlo (MCMC) method within a Bayesian framework was used to estimate the Bayesian posterior probabilities (BPP) of the phylogenetic trees using 50% majority rule (Larget and Simon, 1999). Maximum likelihood (ML) trees were constructed using the RAxMLGUI 1.1 software (Silvestro and Michalak, 2012) with the same nucleotide substitution model as in the BI in 1000 bootstrap replicates for both datasets. The BPP and ML bootstrap (BS) values greater than 50% are assigned for the appropriate clades in BPP/BS pattern. The consensus trees were selected to represent the phylogenetic relationships with branch length and support level. They were visualized using Dendroscope V.3.2.8 (Huson and Scornavacca, 2012) and redrawn in Adobe^®^ Photoshop^®^ 7.0 ME.

## Results

lsid:zoobank.org:pub:B816EC15-1639-4026-91DD-B368876A6946.

**Figure 1:**
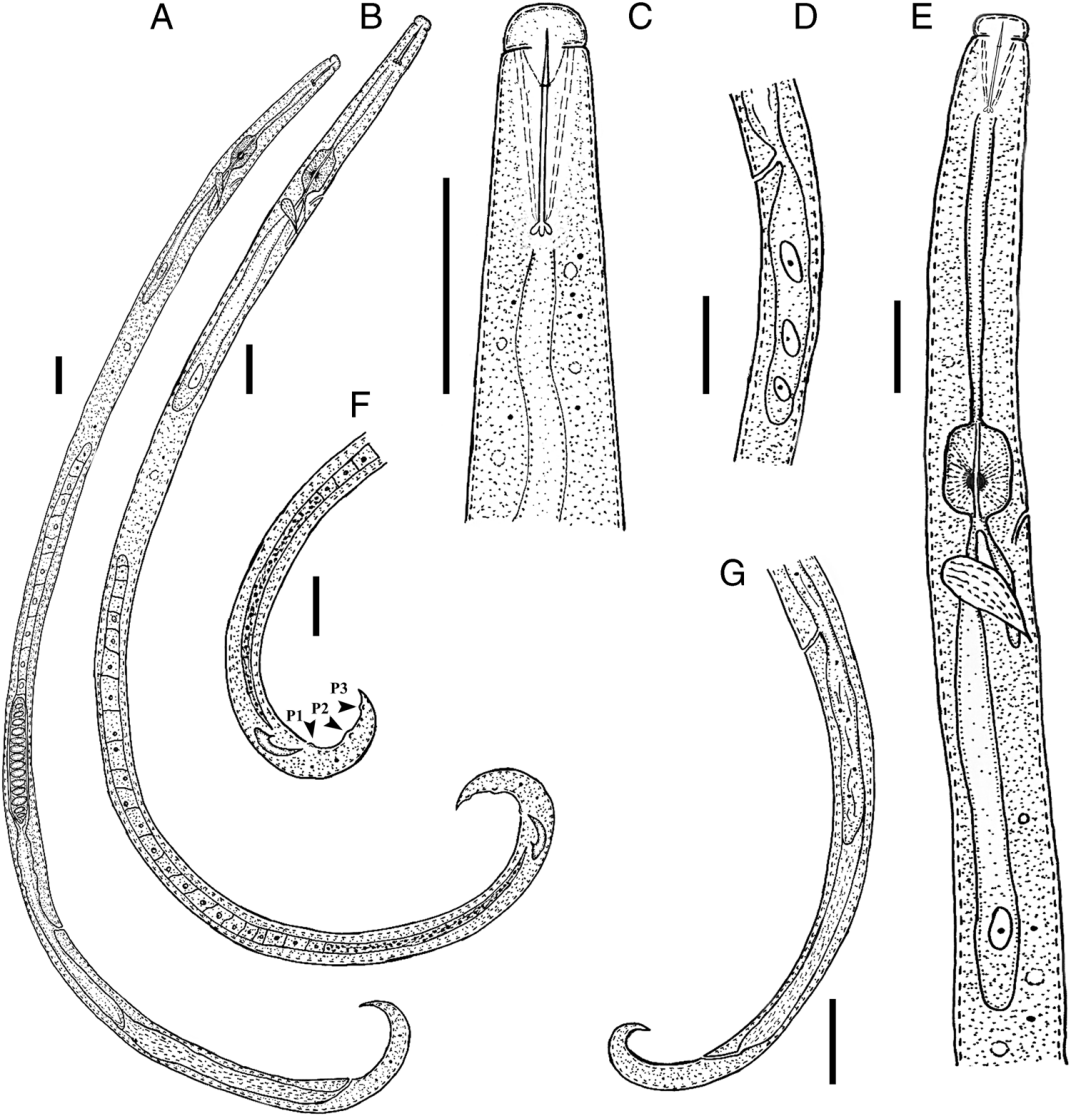
Line drawing of *Basilaphelenchus hyrcanus* n. sp. (A) Female entire body – (B) Male entire body – (C) Female head – (D) Vulval region – (E) Anterior body – (F) Male posterior region in lateral view showing genital papillae (P2-P4) − (G) Female posterior region (Scale bars: A-C and E, F = 10 μm; D, G = 20 μm.)

**Figure 2:**
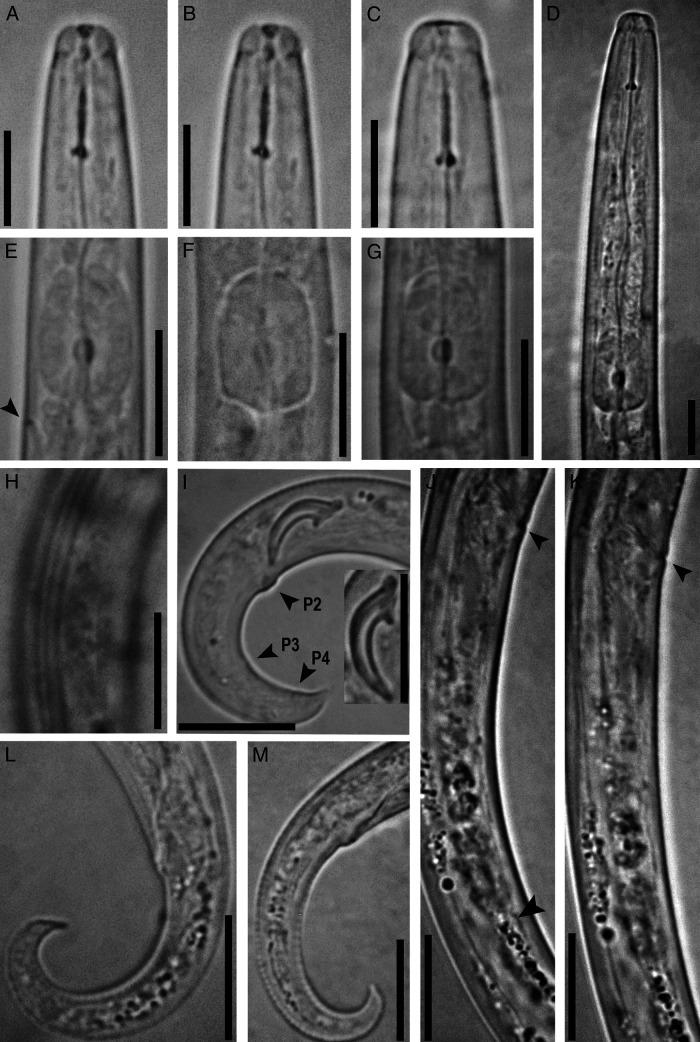
Photomicrographs of *Basilaphelenchus hyrcanus* n. sp. (A-C) Female anterior body – (D) Part of pharynx– (E-G) Metacorpus region showing excretory pore (arrowhead) – (H) Lateral field – (I) Male posterior body showing spicules and papillae arrangement (P2 + P4 arrowheads) – (J, K) Vulval region showing post-vulval uterine sac (arrowhead) – (L, M) Female tail (Scale bars = 10 μm.)

***Basilaphelenchus hyrcanus*** (The species epithet refers to Hyrcania, the ancient Greek name of Golestan, from which the new species was recovered.) **n. sp.** (Figures 1, 2).

### Measurements

Specific measurements are provided in Table 1.

**Table 1.** Morphometric data for *Basilaphelenchus hyrcanus* n. sp. (measurements μm; mean ± s.d. and (range) for paratypes).

	Female		Male
Character	Holotype	Paratypes	Paratypes
n	–	10	7
L	350	383 ± 34.0 (316–418)	370 ± 15.1 (350–395)
a	32.0	37.0 ± 3.5 (31.6–41.8)	40.0 ± 2.2 (37.4–44.0)
b	7.6	8.3 ± 0.7 (7.3–9.5)	7.3 ± 0.2 (7.0–7.6)
b′	3.6	4.0 ± 0.2 (3.5–4.1)	3.6 ± 0.3 (3.3–4.2)
c	12.1	13.0 ± 0.9 (11.5–14.0)	13.6 ± 0.5 (12.8–14.2)
c′	4.8	5.4 ± 0.5 (4.8–6.2)	3.8 ± 0.4 (3.1–4.3)
Vc or T	71.4	69.7 ± 3 (63.0–75.5)	57.3 ± 3.1 (53.5–60.8)
Lip region height	2.0	2.0 ± 0.2 (2.0–2.5)	2.1 ± 0.2 (2.0–2.5)
Lip region width	5.0	4.8 ± 0.4 (4.0–5.0)	4.9 ± 0.4 (4.0–5.0)
Stylet length	9.0	9.4 ± 0.5 (9.0–10.0)	9.3 ± 0.5 (9.0–10.0)
Conus length	4.0	3.4 ± 0.5 (3.0–4.0)	3.4 ± 0.5 (3.0–4.0)
m^1^	44.0	36.0 ± 3.4 (34.0–40.0)	36.0 ± 2.7 (34.0–44.0)
Maximum body diam.	11.0	10.3 ± 0.5 (10.0–11.0)	9.3 ± 0.5 (9.0–10.0)
MB^2^	80.0	85.0 ± 1.8 (83.3–88.0)	86.5 ± 1.3 (84.6–88.2)
Median bulb width	6.0	6.2 ± 0.6 (5.0–7.0)	6.3 ± 0.5 (6.0–7.0)
Median bulb length	10.0	10.7 ± 0.7 (10.0–12.0)	11.1 ± 0.4 (11.0–12.0)
Median bulb length/diam. Ratio	1.7	1.7 ± 0.1 (1.6–2.0)	1.8 ± 0.2 (1.6–2.0)
Nerve ring from anterior body	54.0	51.1 ± 4.1 (45.0–55.0)	56.0 ± 1.8 (55.0–60.0)
Excretory pore from anterior end	44.0	44.1 ± 2.8 (40.0–48.0)	48.6 ± 1.3 (46.0–50.0)
Ovary or Testis length	130	138 ± 10.3 (125–155)	212 ± 16.3 (190–240)
Post-uterine sac	42.0	45.3 ± 7.0 (38.0–60.0)	–
Vulva to anus distance	88.0	89.0 ± 9.2 (68.0–98.0)	–
Post-uterine sac length/vulva to anus (%)	47.7	51.4 ± 9.3 (42.1–70.6)	–
Vulval body diam.	10.0	9.3 ± 0.7 (8.0–10.0)	–
Anal (cloacal) body diam.	6.0	5.6 ± 0.5 (5.0–6.0)	7.3 ± 0.5 (7.0–8.0)
Tail length	29.0	30.0 ± 1.1 (28.0–31.0)	27.3 ± 2.0 (25.0–30.0)
Spicule length (arc)	–	–	10.3 ± 0.5 (10.0–11.0)

Note: ^1^Length of conus as percentage of total stylet length. ^2^Distance between anterior end of body and center of median pharyngeal bulb as percentage of pharyngeal length.

#### Female

Body short, slender, ventrally curved after fixation, more curved near tail region. Cuticle weakly annulated, lateral field with three incisures (i.e., two ridges), not areolated. Lip region separated from rest of body by a shallow, but clear constriction, *ca* 2.1 μm high and 4.8 μm broad. Stylet fine, thin, and slender, with three elongate, posteriorly directed knobs, visible in fresh individuals in temporary mounts in water. Procorpus cylindrical, *ca* 2.5 stylet length long. Median bulb (metacorpus) spherical to rectangular, with glandular anterior third and posterior two-thirds muscular, valve apparatus developed, sclerotised, posteriorly located at 60.0–66.8% of metacorpus length from anterior end of metacorpus. Dorsal pharyngeal gland orifice opening into lumen of metacorpus mid-way between anterior end of metacorpal valve and anterior end of metacorpus. Pharyngo-intestinal junction immediately posterior to metacorpus. Excretory pore located at level of base of metacorpus. Hemizonid not seen. Nerve ring situated at *ca* one metacorpus (median bulb) length posterior to bulb. Pharyngeal glands dorsally overlapping intestine for 47–67 μm, gland margins and nuclei not well discerned. Reproductive system monodelphic, prodelphic, 30–41% total body length long (excluding post-vulval uterine sac (PUS)), comprising ovary, oviduct, spermatheca, crustaformeria, uterus, vagina + vulva and PUS. Ovary single, anteriorly outstretched with oocytes in a single row. Tube-like oviduct connecting ovary and crustaformeria, spermatheca elongate, *ca* 4 body diam. long, filled with spheroid or packed sperm cells, crustaformeria and uterus not clearly discernible, vagina not sclerotised, slightly inclined anteriorly. Vulva simple, without any type of differentiation. PUS *ca* five vulval body diam. long, extending for *ca* 51% of vulva to anus distance. Rectum and anus clearly visible. Tail elongate conical, ventrally bent in distal part, with sharp to finely rounded tip.

#### Male

Body cylindrical, J-shaped when heat-relaxed. Cuticle and anterior region similar to female. Gonad to right of intestine, outstretched with developing spermatocytes in a single row. *Vas deferens* composed of small rounded cells, merging with distal part of intestine to form a simple tube connected to cloacal opening. Spicules small, separate, arcuate, with bluntly rounded condylus, rostrum short and pointed, distal ends of spicules with pointed tip. Bursa or bursal flap apparatus and gubernaculum absent. No single precloacal papilla (P1) observed. Three pairs of subventral caudal papillae present arranged as follows: first pair located just posterior to cloacal aperture (P2), second pair of postcloacal subventral papillae (P3) located at *ca* 53% of tail length from cloacal slit, last postcloacal pair located just anterior to tail end (P4). Tail conical, with sharp or finely rounded tip or small mucron like projection.

### Type host and locality

The new species was recovered from bark samples of a beech tree (*Fagus orientalis*) collected from Aliabad-e-Katul, Golestan Province, northern Iran, in 2019 (GPS coordinates: 36°53ʹ33.37ʺN, 54°50ʹ43.19ʺE, 159 m a.s.l.).

### Type material

Holotype female (slide ABH001) together with ten paratype specimens (six females and four males; slides ABH001, ABH002) deposited in the Nematode Collection of the Department of Plant Protection, College of Agriculture and Natural Resources, University of Tehran, Karaj, Iran. Three paratype females and two paratype males deposited at Wageningen Nematode Collection, the Netherlands, and two paratype females and a paratype male deposited in the National Nematode Collection of the Department of Nematology, Iranian Research Institute of Plant Protection, Tehran, Iran.

### Diagnosis and relationships

*Basilaphelenchus hyrcanus* n. sp. is characterized by a body length of 383 (316–418) μm in females and 370 (350–395) μm in males, elevated lip region with sclerotised vestibule and cephalic framework, stylet 9.3 (9.0–10.0) μm long with three elongate, posteriorly directed knobs, median bulb well developed, elongate, filling corresponding body region with developed postmedian valve, excretory pore located at level of base of metacorpus, PUS 38–60 μm long, female tail ventrally bent, conoid, elongate with sharp to finely rounded tip, and males with small, well curved spicules, three pairs of small papilla-shaped caudal papillae, and no bursa at tail tip.

*Basilaphelenchus hyrcanus* represents the sixth species of the genus and was compared with all other known species of the genus. The new species belongs to *Basilaphelenchus* in having the genus diagnostic characters such as stylet and body shape and tail characters, as well by the SSU and LSU markers, and can be separated from the five previously described species by the longer stylet. The detailed comparisons are as follows: the new species can be distinguished from *B. persicus* by slightly longer body length of 383 (316–416) vs 352 (297–393) μm, c = 13 (11.5–14) vs 9.7 (8.3–11.8), longer stylet of 9.4 (9.0–10.0) vs 6.7 (5.5–7.5) μm, metacorpus shape (well developed, elongate, filling corresponding body region with developed valve *vs* small, spherical, valve weak), and slightly shorter tail length of 30 (28–31) vs 36 (29–45) μm; from *B. brevicaudatus* by longer stylet length of 9.4 (9.0–10.0) vs 6.4 (6–7) μm, c = 13 (11.5–14) vs 22.5 (19.5–26.6), c′ = 5.4 (4.8–6.2) vs 2.6 (1.9–3.3), longer PUS of 45.3 (38–60) vs 32.4 (29–37) μm, metacorpus shape (well developed, elongate, filling corresponding body region with developed valve vs small, spherical, valve weak), and female tail characters (conical, gradually narrowing, ventrally bent, 30 (28–31) μm long vs short conical, 20 (17–24) μm long); from *B. gorganensis* by female tail characters (conical, gradually narrowing, ventrally bent, 30 (28–31) μm long vs conical, dorsally convex, ventrally concave, its tip sharp, 24 (22–27) μm long), slightly shorter body length of 383 (316–416) vs 481 (415–559) μm, longer stylet of 9.4 (9.0–10.0) vs 6.2 (5.6–7) μm, c = 13 (11.5–14) vs 20 (18–24), shorter PUS of 45.3 (38–60) vs 68 (59–79) μm, and metacorpus shape (well developed, elongate, filling corresponding body region with developed valve *vs* small, spherical, valve weak); from *B. magnabulbus* by longer stylet of 9.4 (9.0–10.0) vs 6.6 (6–7.5) μm, position of the excretory pore (at level of base of metacorpus *vs* posterior to base of metacorpus), and male tail tip characters (sharp or finely rounded tip or small mucron like projection vs bluntly or finely rounded); and from *B. grosmannae* (Rühm, 1965) Pedram et al., 2018, by c = 13 (11.5–14) vs (16–18.7), a = 37 (31.6–41.8) vs (29–30.6), b = 8.3 (7.3–9.5) vs (6.5–7.1), and longer tail length of 30 (28–31) vs *ca* 24.5 μm.

### Bionomics

Specimens of *B. hyrcanus* n. sp. were successfully multiplied on a *B. cinerea* culture.

### Molecular phylogeny

**Figure 3:**
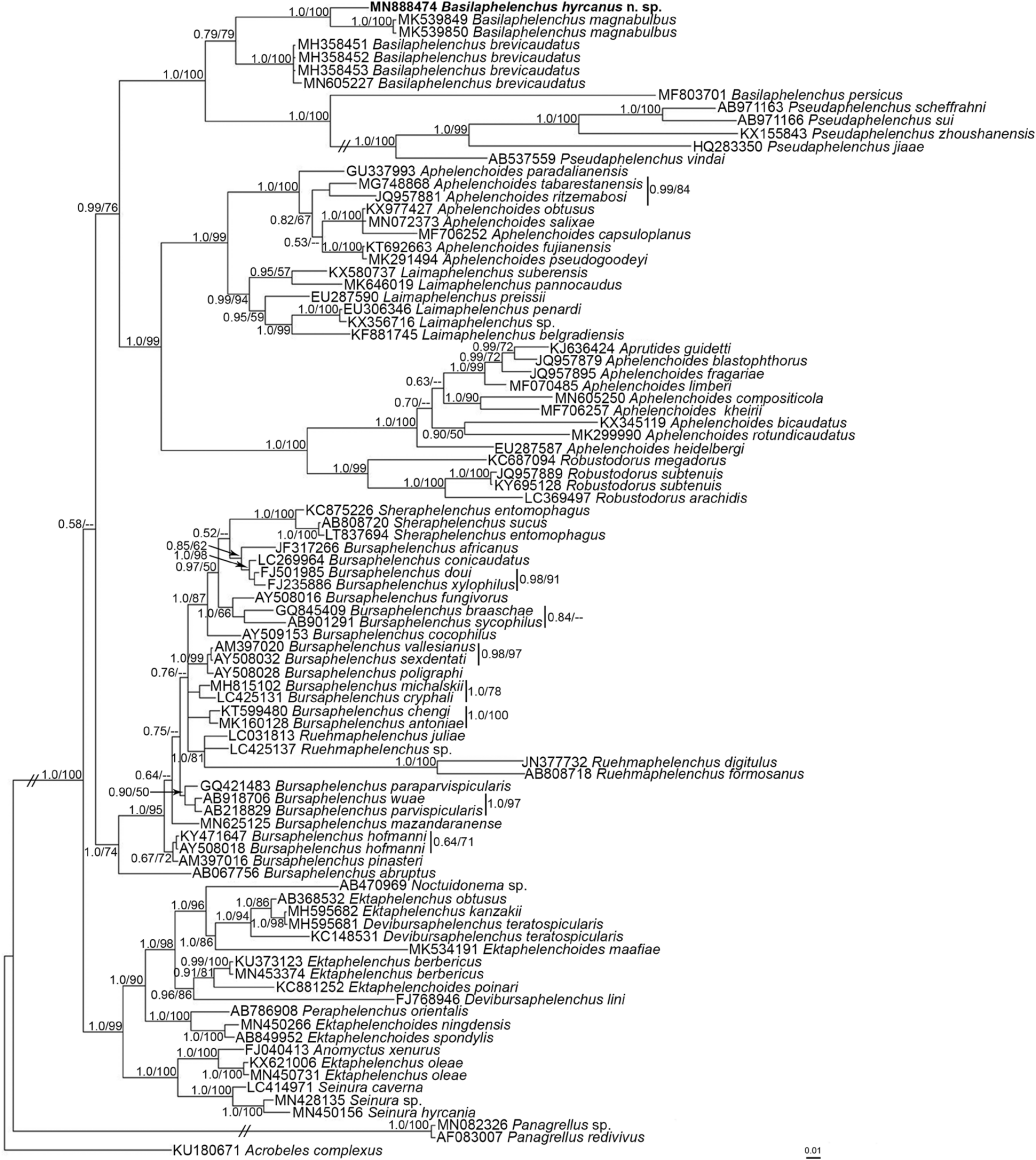
Bayesian 50% majority rule consensus tree inferred from the small subunit (SSU) rDNA gene sequences of *Basilaphelenchus hyrcanus* n. sp. under the GTR + G + I model. Bayesian posterior probabilities (BPP) and maximum likelihood bootstrap (ML BS) values greater than 0.50 and 50, respectively, are given for appropriate clades in the pattern of BPP/ML BS. The new species taxon is represented in bold.

**Figure 4:**
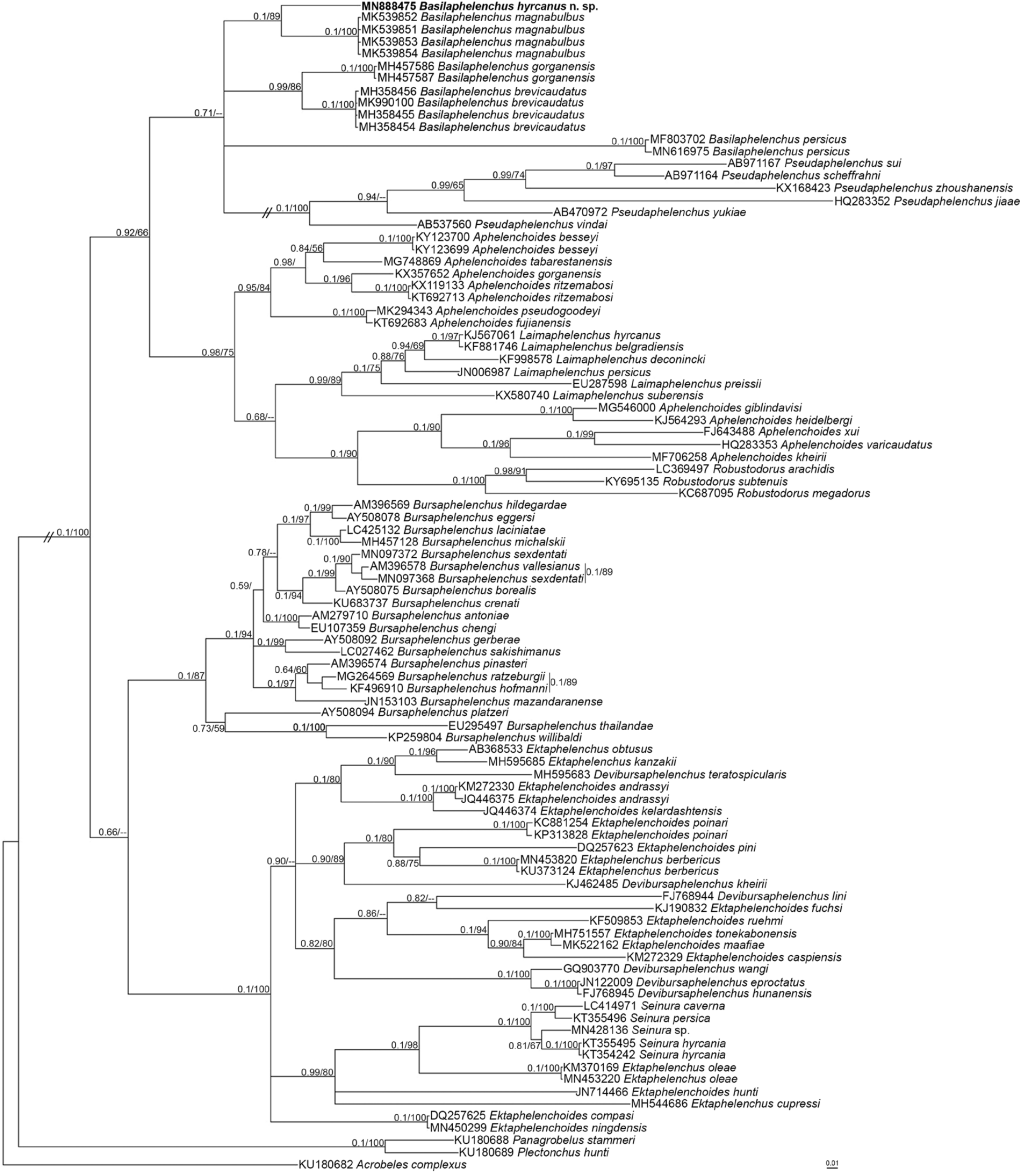
Bayesian 50% majority rule consensus tree inferred from the D2-D3 large subunit (LSU) rDNA gene sequences of *Basilaphelenchus hyrcanus* n. sp. under the GTR + G + I model. Bayesian posterior probabilities (BPP) and maximum likelihood bootstrap (ML BS) values greater than 0.50 and 50, respectively, are given for appropriate clades in the pattern of BPP/ML BS. The new species taxon is represented in bold.

Partial sequences of the 18 S region and D2-D3 28 S expansion segments of the rDNA gene were generated with accession numbers MN888474 (730 bp) and MN888475 (718 bp), respectively. The datasets for phylogenetic trees were composed of 1,529 and 975 total characters in 18 S and D2-D3 28 S, respectively, of which 867 and 718 characters were variable after aligning with MAFFT and manually editing. In both datasets, members of Panagrolaimoidea and Cephaloboidea were used as outgroups, the taxa which are usually used in related studies (e.g., Aliramaji et al., 2019a; Kanzaki et al., 2014; Mirzaie Fouladvand et al., 2019a, b). Both BI and ML approaches under the GTR + I + G model were used for the phylogenetic study on aphelenchid isolates in both datasets (Figures 3 and 4).

Members of *Basilaphelenchus* and *Pseudaphelenchus*, the only two genera of Tylaphelenchinae that have sequences available in NCBI, clustered into a monophyletic group based on both the 18 S and D2-D3 28 S genes in concordance with the related studies (Aliramaji et al., 2019a; Mirzaie Fouladvand et al., 2019a, b; Pedram et al., 2018). *Basilaphelenchus hyrcanus* n. sp. formed a well-supported molecular clade with isolates of *B. magnabulbus*, the species showing the greatest morphological resemblance. A pairwise sequence alignment comparison between the new species and the other species of the genus showed 4.4–8.4% (33–61 bp) and 13.3–19.5% (96–134 bp) sequence divergence in 18 S and D2-D3 28 S, respectively, which is more than expected with respect to the great similarity in morphology of *Basilaphelenchus* spp. Morphological resemblance among *Basilaphelenchus* spp. suggests that a molecular approach is strictly necessary for identification and to reveal the existence of any cryptic species.

## Discussion

Considering the current study and transition of *Tylaphelenchus grosmannae* Rühm, 1965 to *Basilaphelenchus* by Pedram et al. (2018), the newly established genus now comprises six nominal species. The other newly described species were recovered from the northern Province in Iran in recent years (Aliramaji et al., 2019a; Mirzaie Fouladvand et al., 2019a, b; Pedram et al., 2018). The habitat of the genus seems to be in association with wood borer and bark beetle insects as all the species were found in rotten wood and bark samples. Mycetophagus habit was also proposed for genus members due to successful multiplication of the type species on fungus (Pedram et al., 2018) and the locations where the genus members have been recovered (Mirzaie Fouladvand et al., 2019a). *Basilaphelenchus hyrcanus* n. sp. was also successfully multiplied on fungus medium in this study. In addition, *Basilaphelenchus* cf. *gorganensis* multiplication on fungus medium (on *Botrytis* sp.) was observed under laboratory conditions (Unpublished data). Multiplication of three species out of six nominal species on fungus medium strongly supports mycophagy hypothesis for this genus. Moreover, the other aphelenchid genera described in association with insects are considered to have either predatory habits as in *Ektaphelenchus* Fuchs, 1937 (Golhasan et al., 2019a; Gu et al., 2013; Miraeiz et al., 2017) and *Ektaphelenchoides* Baujard, 1984 (Golhasan et al., 2019b; Kanzaki, 2014) or mycophagy in *Cryptaphelenchus* Fuchs, 1937 (Aliramaji et al., 2019b). It remains to be determined whether mycophagous *Cryptaphelenchus* species have insect associations similar to those of *Basilaphelenchus*.

Members of the genus *Basilaphelenchus* show a great divergence in molecular characters based on two rDNA markers, especially D2-D3 28 S expansion segments, although the conventional morphological and morphometrical traits do not reflect these large differences. This inconsistency resulted in the use of a reverse taxonomy approach in purpose of generic and species level identification. So a molecular approach superseded traditional morphological identification for the genus in this study. However, there are a few morphological and morphometrical characters that still remain efficient for clear comparison purposes. According to all described *Basilaphelenchus* species, tail characters including length (c and c´ indices), shape and terminus feature together with adult body length are the most important features for differentiation. On a second level, PUS length, vulva position (V index) and metacorpus shape can be considered for intrageneric identification. Meanwhile there are some features that are stable diagnostic characters for the genus. Stylet knobs shape, metacorpus valve position, lateral lines, number and position of male papillae and spicules structure are remarkably constant at the generic level. Hence, SEM study of head structure may supplement diagnostic character for the genus (Davies et al., 2015; Golhasan et al., 2019a, b; Miraeiz et al., 2017).

Monophyly of Tylaphelenchinae members excluding the genera *Albiziaphelenchus* and *Tylaphelenchus* have been supported using both large and small ribosomal subunits markers (Mirzaie Fouladvand et al., 2019a, b; Pedram et al., 2018) and in our molecular analysis. However, differentiation between *Basilaphelenchus* spp. and *Tylaphelenchus* spp. is still doubtful due to the great resemblance in main morphological features and lack of molecular data. Therefore, improving the poor description of *Tylaphelenchus* populations through examination of the existing type materials, their molecular characters, or by recovering specimens from the original localities are recommended.
